# Accelerating Learning Health Systems Using Alicanto Collaboration Platforms

**DOI:** 10.1200/GO.22.68000

**Published:** 2022-05-05

**Authors:** Yuri Quintana, David Einstein, Robin Joyce, Andrew Lyu, Nuha El Sayed, Carolyn Andrews, Tim Rebbeck

**Affiliations:** ^1^ Beth Israel Deaconess Medical Center, Boston, MA; ^2^ Joslin Diabetes, Boston, MA; ^3^ Dana-Farber Cancer Institute, Boston, MA

## Abstract

Global health challenges require systematic approaches that bring together a wide range of stakeholders and experts unevenly distributed worldwide. We saw an even greater need to find novel ways to collaborate during the pandemic due to limited in-person meetings and travel restrictions. The purpose of this project is to accelerate collaborations in learning health systems with online technologies to improve communication, education, and knowledge management.

**METHODS:**

We developed Alicanto Cloud, an online collaboration platform for healthcare professionals to support learning health systems. Alicanto aims to help groups communicate, share educational resources, discuss complex clinical cases across institutions and countries. Alicanto provides multiple ways for users to create synchronous and asynchronous discussions. Alicanto was developed by the BIDMC Division of Clinical Informatics in collaboration with local and international colleagues with a specific focus on global health and capacity building.

**RESULTS:**

Alicanto is being used worldwide for cancer, pediatrics, and diabetes collaborations. Online communities use Alicanto to standardize care guidelines, disseminate clinical care best practices, provide online training, conduct virtual tumor boards, and improve knowledge management. Beth Israel Deaconess Medical Center has used Alicanto to connect 13 hospitals and has completed over 600 virtual tumor board case discussions. Dana-Farber Cancer Institute uses Alicanto as an online cancer training network for sub-Saharan Africa. Doctors from Joslin Clinic have used Alicanto to create a global diabetes education network. We will discuss lessons learned in implementing these networks and best practices for accelerating the process of evidence synthesis to dissemination and capacity building.

**CONCLUSION:**

Communities of practice can accelerate their communications and collaborations with the appropriate tools and platforms tailored to their network needs. Further research is needed to understand the optimal ways to create sustained growth and support of communities of practice in learning health systems.

